# Effect of family life quality on youth badminton athletes' identity and achievement

**DOI:** 10.3389/fpsyg.2025.1559107

**Published:** 2025-04-08

**Authors:** Dan Zhu, Ye-Jun Li, Chang-Fa Tang

**Affiliations:** ^1^College of Physical Education, Hunan Normal University, Changsha, China; ^2^School of Educational Science, Hunan Normal University, Changsha, China; ^3^Department of Physical Education and Research, Hunan Institute of Technology, Hengyang, China

**Keywords:** adolescent athletes, quality of family life, athlete identity, athlete achievement, badminton athletes

## Abstract

**Introduction:**

Young badminton players who want to achieve certain achievements are faced with inevitable material and mental pressure. Adolescent families can effectively relieve these pressures for them. A large number of studies have demonstrated the influence of family on athlete achievement, but the specific pathways are not clear. The purpose of this study was to explore the impact of family quality of life on the achievement of youth badminton athletes from the perspective of athlete identity.

**Methods:**

A total of 111 badminton athletes aged 14–18 (M = 16.27, SD = 1.22) in high school were surveyed using the Athlete Identity Measurement Scale (AIMS) and the Family Quality of Life Scale (FQOL).

**Results:**

The results show that family quality of life can positively affect athlete achievement. Those athletes who have achieved athletic success generally have better family economic conditions and a harmonious atmosphere of family interaction, but there is a common problem of insufficient psychological care. Secondly, the quality of family life also affects athlete identity. However, there was no significant correlation between the negative affective dimensions in athletes' identification. Third, athlete identity plays a mediating role between family quality of life and athlete achievement.

**Discussion:**

Overall, family quality of life is associated with athlete achievement. This study found that the intangible psychological and spiritual care and support of badminton players' families lagged behind the tangible material support. In other words, the family's dedication and support for badminton players should start from the athlete's identity and increase the care at the spiritual level. In addition, there are still some factors that have nothing to do with the quality of life of the family, which need to be dealt with by the athletes themselves. Therefore, parents do not need to be overly anxious about the athletic achievements of their teens.

## 1 Introduction

In November 2024, the General Administration of Sport of China (SGAS) issued the “Athlete Technical Grade Standard”. Compared with the previous version of the badminton player technical grade standard, an obvious change of this version is that team athletes have at least one victory record in order to obtain the athlete certificate of the corresponding level of the team (Department of competitive sports, 2024). This means that it is more difficult for youth badminton athletes to obtain grade certificates, so they have to have to increase their training time and intensity. These young athletes who want to excel in their dual careers as school students and athletes require particular types of support within their home, at school, and in their sport context (Knight et al., 2018).

Family sports culture and family support have an important influence on the continued participation of adolescents in sports. For some adolescents, continued participation in sports in late adolescence may require more support from parents, which implies a stronger reliance on a sport-friendly family culture (Strandbu et al., 2019). In addition, studies have shown that family socio-economic status (parents' educational level, parents' occupation, and family income) affects children's academic achievement (Zhang et al., 2020). Therefore, for athletes, family economic and social status should also affect their sports achievements. To be specific, individual training of badminton consumables, equipment and clothing, field fees, training fees, participation fees, etc. are not small expenses, especially with the improvement of badminton level, these costs also increase. In addition, finding the right coach according to the competitive level of athletes at different stages also needs to use the social resources of the family, which requires the full understanding and support of the family, and needs to be put into action. So the difficulty of the badminton grade certificate seems to be a requirement for the athletes, but it is actually a new round of adjustment for the athletes' families. Corresponding adjustments should be made in various aspects such as economic expenditure, emotional companionship and social resources. Gradually, it's became an common phenomena in China that all family members are involved in children' training and competition, and some parents even choose to give up their own work and social life. They regard their children's athletic performance as their career. An individual's identity cannot exist in isolation from his or her background culture and society (Kidwell et al., 1995). The excessive attention and investment of family members led by parents make the achievements of young badminton players have to be affected by these factors. This kind of family culture and social atmosphere influences the athlete's identity, which can play a role in the athlete's performance. Compared with athletes with low athlete identity, athletes with high athlete identity performed better in concentration, confidence and achievement motivation, goal setting and mental preparation, and peaking under pressure (Christino et al., 2024). Therefore, it is very important to study how family influences the achievement of young badminton players for their mental health and performance.

Since family plays an important role in the success of young badminton players, what kind of support do they need? How much support and help is useful? In fact, the success of elite athletes is not so simple. Athletes with a high quality of family life are not necessarily good at sports. The blind stacking of material economy, emotional companionship and social resources does not always have good feedback. And too much investment also means too much stress, which affects the athlete's self-identity and performance, even if the parents do not emphasize those investments (Knight et al., 2010). Sometimes, parents don't need to put too much emphasis on the importance of their investment. Studies have found that children whose parents are not involved in sports are also able to get high levels of athletic performance (Kovács et al., 2024). Whether the support and effort of the family can be rewarded depends on the attitude of the athletes themselves how to view these supplies, which means that these support into positive or negative energy. From the perspective of identity theory, family and social environment can enrich individual roles, but ultimately, role identification depends on individual acceptance (Stets and Burke, 2000). Athlete identity can exist as a role-based self-concept with perceived expectations related to the performance of that role (Stets and Burke, 2000), such as accepting the role expected by parents, coaches, and peers, and acting accordingly. That said, the amount of support a family is able to provide is less important than the extent to which athletes see it as supportive, which will directly affect their psychosocial outcomes and athletic performance (Babkes and Weiss, 1999). Moreover, individual psychological motivation will effectively guide and determine individual behavior, and the results formed by behavioral practice will often change, influence or determine the form of thinking, methods and the subject's experience and emotion. Although teen athletes want their parents involved in their sports. They also wanted encouragement from parents, especially when they were feeling down, but they wanted to limit the ways in which parents could participate in sports. In addition, parents are asked not to interfere with athletes' social relationships, and the final decision on whether to participate in sports should be made by them (Strandbu et al., 2017). That is to say, although athletes need parental involvement and support to some extent, their true inner motivation at other levels is inconsistent with their apparent needs. Therefore, looking at family support from the perspective of athlete psychology can effectively answer the question “What should family support?” And “How to support the question?”. This study is based on athlete identity to analyze the influence of family life quality factors on athlete achievement.

Numerous studies have demonstrated the importance of family support for athletic performance (Zhang et al., 2020), while athlete self-identity is also closely related to athlete achievement (Edison et al., 2021). However, there are few studies on the relationship between family life quality, athlete identity and athlete achievement. In fact, the prediction of athlete identity contributes to a better understanding of the effect of family life quality in sports. Based on social identity theory and social cognition theory, this article introduces the variable of athlete identity to explore the influence of family life quality on athlete achievement. The analysis of family life quality from the perspective of athletes has more reference value, which is related to the development trend of Chinese parenting. Are athletes' achievements positively correlated with their family's quality of life? Does the quality of family life affect athlete identity? Is it necessary for the whole family to accompany athletes to training? Therefore, this study explores the influence of family life quality on Chinese athletes' identity and performance, so as to determine what kind of family support badminton players need. Its value is to promote the mental health of young athletes, provide suggestions for family athlete training, and relieve the anxiety of overstressed athlete parents.

### 1.1 Empirical background

Many studies have proved that youth participation in sports activities cannot be separated from family support, especially for athletes. Parental support is the core factor of youth physical activity, especially tangible support, but parental control behavior will reduce the intensity of adolescent physical activity (Doggui et al., 2021). In China, parents' physical activity has always been positively correlated with children's physical activity, among which mothers' physical attitude has the greatest impact on children (Lu et al., 2021). Children whose parents are involved in both sports and education tend to win prizes at national sports competitions and are more likely to continue playing regular and competitive sports in the future than students whose parents are not involved (Kovács et al., 2024). Apart from the tangible or intangible support and help of parents in the family, family culture is also one of the important factors affecting the achievement of athletes. Those athletes who are able to participate in sports activities for a long time are mainly influenced by the family sports culture (Strandbu et al., 2019). Parents' role shaping of physical activity and the amount of time adolescents currently spend playing sports significantly affect youth's participation in sports. And parents' sports behavior will have a profound impact on teenagers' sports values (Jaf et al., 2021). In short, family support for athletes is mainly reflected in the following aspects: (a)economic basis; (b)emotional companionship; (c) social resources (Dong et al., 2023); (d) family sports culture.

Gender factors should be taken into account when studying the link between family support and achievement in young athletes. Studies have shown that men are more likely than women to receive family support, especially in sports. In the context of physical activity, men are usually the dominant group, although female participation has increased (Heinze et al., 2017). As a result, parents generally believe that sporting achievement increases boys' status and popularity more definitively than that of girls, for which they are willing to invest more (Heinze et al., 2017). In other words, male athletes are more likely to receive family support than female athletes, which may provide an analytical perspective for the achievement of male athletes.

Athlete identity is the bridge between family life quality and athlete achievement. Athletic identity refers to the degree of strength and exclusivity to which a person identifies with the athlete role or the degree to which one devotes special attention to sport relative to other engagements or activities in life (Brewer et al., 1993). Based on psychological and social identity, the role or identity of an athlete can affect an individual's self-esteem, motivation, and development prospects, mainly through athletic ability, performance, and perceptions of achievement (Stets and Burke, 2000). Athletes with strong athlete identity are more likely to be depressed than those with moderate and low athlete identity. Female athletes generally have lower sports identification than male athletes, but their anxiety level is higher than that of male athletes (Antoniak et al., 2022). Therefore, for such badminton players, their families should give them more spiritual care and pay attention to their anxiety, especially the families of female athletes. Too strong individual athletic identity can lead to extreme performance-enhancing strategy selection (Stets and Burke, 2000), overtraining (Coker-Cranney et al., 2018), sports injury and disordered eating patterns. In short, the impact of athlete's identity on their achievements is more intuitive, it directly affects the athlete's choice of skills and tactics, diet adjustment, emotional state and other aspects directly related to training or competition.

### 1.2 Theoretical underpinnings

#### 1.2.1 Social identity theory

Identity is an individual's subjective and internal self-concept, a sense of belonging to a particular group, and the values and emotions that an individual perceives as a member of that group (Ashforth and Mael, 1989). Social Identity Theory (SIT) provides a social-psychological perspective that can be effectively applied to organizational behavior (Neighbors et al., 2013). Social identity is a self-definition that explicitly guides how to conceptualize and evaluate oneself. Social identity includes many unique and distinctive characteristics of oneself, such as gender, occupation, interests, political parties and religious alliances. Also, athletes can achieve a kind of social identity according to the training characteristics of the common sports. In the social group of teenagers, athletes should be more influenced by other athletes (Neighbors et al., 2013). When a person identifies more closely with that group, identification with others enhances their influence over themselves.

#### 1.2.2 Social cognition theory

Social cognitive theory is a theory that adds cognitive components to traditional behaviorist personality theory to explain the process of individuals in social learning (Bandura, 1982)^.^ Social cognitive theory generally brings together three aspects: (a) Reciprocal Determinism; (b) Observation Learning; (c) Self-efficacy. Interactive determinism emphasizes the interaction between people and the external environment, and the external environment is not only the cause of individual behavior, but also the result of individual behavior (Bandura, 1982). An athlete's family support is not only the reason why he or she strives for athletic performance, but also the result of his or her own achievements. Observational learning is the process by which an individual acquires or improves certain skills through the vicarious experience of observing the behavior of others. From this perspective, among the many family factors that affect athletes' physical exercise behavior, the exercise habits observed by their parents and the sportsmanship they feel will become a kind of surrogate experience for individual behavior acquisition (ASukys et al., 2017), so that teenagers can acquire relevant sports cognition through observation and attention, learn certain sports skills and form certain exercise behaviors. Self-efficacy is an individual's self-judgment of the effectiveness of the interaction between himself and the environment. A strong sense of self-efficacy helps an individual to have interest in new problems and devote himself to them, which helps to motivate an individual to constantly strive to overcome difficulties. Among them, the positive self-efficacy of individuals brings faith while negative emotions produce frustration (Edison et al., 2021). The positive self-efficacy brought by family support can make athletes produce affirmative emotions to cope with setbacks and injuries in training.

Based on the above empirical analysis and theoretical explanation, combined with the main research purpose of this article, the following hypotheses were designed to explore the impact of family life quality on the achievement of young athletes.

Hypothesis 1 (H_1_): Family life quality positively influences the achievement of young badminton athletes.

Hypothesis 2 (H_2_): Athlete identity mediates the relationship between family life quality and badminton athletic achievement of young athletes.

Hypothesis 3 (H_3_): The quality of family life positively affects the identity of young badminton athletes.

**Figure 1:**
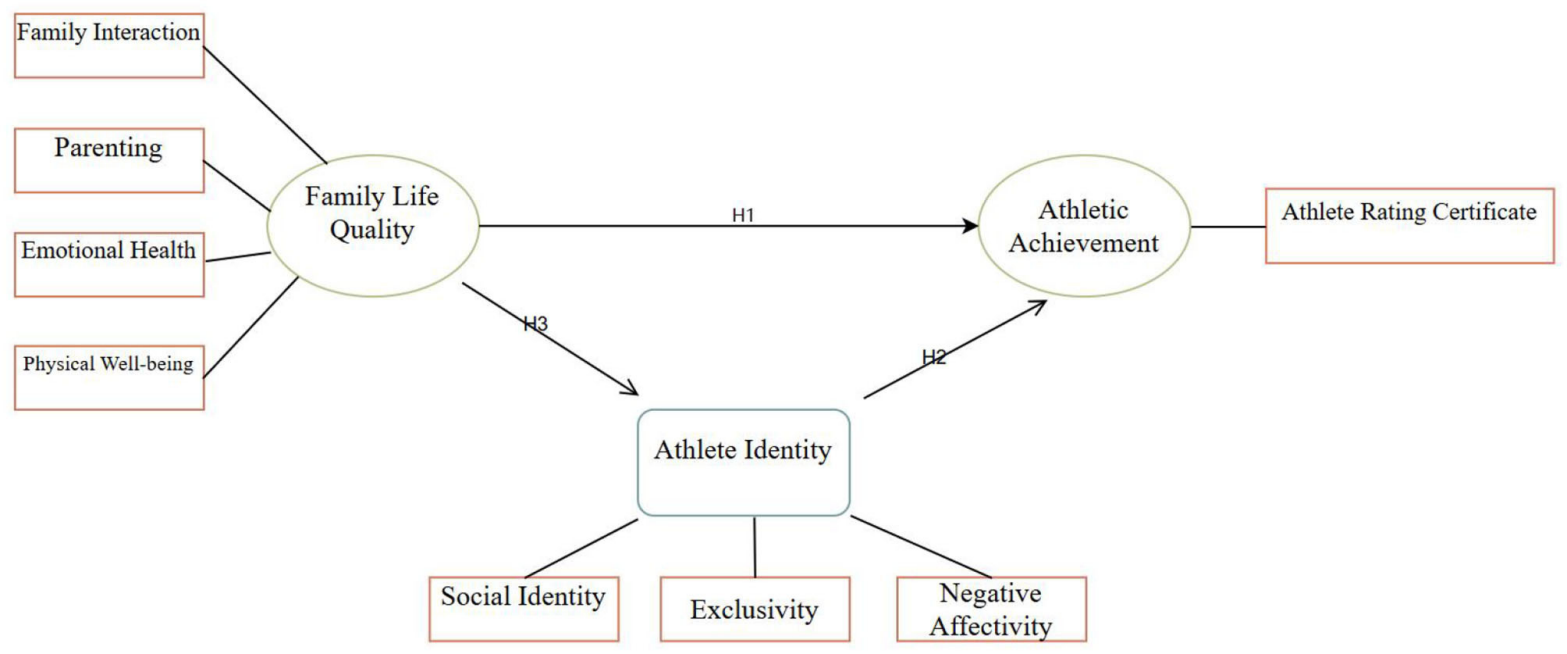
Conceptual framework.

And the conceptual framework of this study is basically clear (Figure 1).

## 2 Method

### 2.1 Procedure

The survey was carried out by convenience sampling. For athletes, the following inclusion criteria were included (a) has been registered with the China Badminton Association; (b) still involved in the badminton training team; (c) be able to represent their region in competitions; (d) attending in a regular high school. The sampling method in this study is the purposive sampling of non-probability sampling. Firstly, young athletes eligible for the study were initially screened, and then the distribution areas of these athletes were determined. Finally, questionnaires were distributed and collected in these areas, and it includes Hengyang, Yiyang, Changsha, Xiangtan and Loudi. On the premise of preliminary communication and approval with the athletes and their coaches, the parents were also informed and the questionnaires were distributed with the permission of the athletes themselves and their guardians. The research was conducted with two forms of online questionnaire system and offline questionnaire filling, the filling out lasted 5–10 min. Before the questionnaire, participants were informed of the goal and main contents of the survey, and were promised that the information they provided would be kept confidential and used only for scientific research. Participants were told that participation in the study was completely voluntary and that they could stop participating at any time. Ultimately, their questionnaires were submitted anonymously.

The first part of the questionnaire contained questions regarding demographic and badminton grade certificates obtained so far. This study mainly takes badminton grade certificate as the explicit reference of athlete achievement. In China, players who have obtained badminton level certificates have one more chance to be admitted to university than ordinary students, and their scores are lower than those of ordinary students. This is also the original intention of most badminton athletes to insist on sports in the junior high school stage when the academic pressure is huge. Physical education is not a priority in the Chinese education system (Downward et al., 2014). Intellectual education is the choice of most parents and students, especially those with excellent grades. The second and third parts of the questionnaire are athlete identity measurement scale (AIMS) and family quality of life scale (FQOL) respectively. The research was conducted in accordance with the Declaration of Helsinki and approved by the Biomedical Research Ethics Committee of Hunan Normal University; with license number 772. Data were collected between November 2024 and December 2024.

### 2.2 Participants

The subjects of this study were strictly screened, and questionnaires were distributed when the conditions were basically met. The participants in this study were mainly from Hunan Province in central China. Many badminton world champions come from Hunan, such as Bao Chunlai, Gong Zhichao, Gong Ruina, Jia Yifan and so on. Hunan has sent a large number of talents for Chinese badminton, and its badminton level is at the forefront of China. Therefore, it is of great reference value to investigate the young athletes in Hunan Province. All the subjects are young badminton athletes who are currently studying in ordinary high schools and participating in training and competitions. The study sample included 115 youth badminton athletes. These high school athletes not only face the pressure of further study, but also face the pressure of badminton competition. Meanwhile, these subjects selected by the research are relatively excellent young athletes who are eager for sports achievements, which is in line with the theme of this study. In addition, the parents of these athletes accompanied them to train almost every day, which provided a reliable source of materials for in-depth interviews and investigations in this study. The study sample included 111 athletes; 1 athletes were excluded due to missing data and 3 athletes were excluded due to invalid responses. It is worth mentioning that registered athletes who are able to represent their region require a high level of badminton, and those who are not likely to win will not be considered for the team. Therefore, there are not too many registered badminton players who meet the above conditions, and the number of athletes who can stick to it will gradually decrease with the growth of age, and the badminton team in a region is generally <40 people. This research also excluded athletes who were too young, so the study sample size was not too large.

At the time of data collection, 63 male and 48 female players were aged between 16 and 17 years (M = 16.27, SD = 1.22). This age range was selected because: Chinese male athletes must be at least 16 years old and female athletes must be at least 15 years old to get the athlete grade certificate. Athletes under the age of 15 were therefore excluded from the sample. The most common athletic achievements at this age fall into three categories: (a) Level I athletes (*n* = 25); (b) Level II athletes (*n* = 22); No athlete certificate (*n* = 64). This study regarded the athlete grade certificate as the evaluation standard of athletes' achievements is because of the value of the certificate. The examination conditions of Chinese badminton athlete grade certificate: (1) Top 8 or top 6 in the national youth competition; (2) Top six or top two teams in provincial cities' competitions.

### 2.3 Measures

Athlete Identity Measurement Scale (AIMS) was used to measure the degree to which an individual identifies with the athlete role (Brewer et al., 1993). The questionnaire consists of seven items in three dimensions: social identity (items 1–3), exclusivity (items 4 and 5), and negative affectivity (items 7 and 8) (Brewer and Cornelius, 2001).

Family Life Quality Scale (FQOL). Athletes' family life quality was assessed though the perceived this questionnaire (Hoffman et al., 2006). The questionnaire included 25 items in five dimensions that examined family quality of life from family interaction, parenting, emotional health, physical wellbeing, and disability-related support, with responses provided on a 5-point Likert scale, ranging from 1 (not at all) to 5 (very much) (Hoffman et al., 2006). The questionnaire was slightly modified to take into account the low prevalence of disability-related support. Finally, the questionnaire is presented with 21 items in four dimensions: family interaction (items 1–6), parenting (items 7–12), emotional health (13–16), physical wellbeing (17–21).

Questionnaires either available in Chinese or translated from English into Chinese using a back-translation procedure as recommended by Hambleton and Zenisky (2010) were used in the present study. The questionnaire used in this study assessed internal consistency using Cronbach's α (Tang et al., 2014)^.^ Alpha values were described as excellent (0.93–0.94), strong (0.91–0.93), reliable (0.84–0.90), robust (0.81), fairly high (0.76–0.95), high (0.73–0.95), good (0.71–0.91), relatively high (0.70–0.77), slightly low (0.68), reasonable (0.67–0.87), adequate (0.64–0.85), moderate (0.61–0.65), satisfactory (0.58–0.97), acceptable (0.45–0.98), sufficient (0.45–0.96), not satisfactory (0.4–0.55) and low (0.11) (Taber, 2018). Each item of the scale was tested, and the results were shown in the Table 1. Furthermore, the general criterion of >0.40 was met by the Corrected Item-Total Correlation (CITC) between the observed variables and their latent variables, indicating that each latent variable's measurement items are appropriately fitted and that the instruments' reliability can be considered reasonable (Hobart and Cano, 2009). The results of Table 1 show that all items of the scale meet the requirements.

**Table 1:** Reliability of all the measures.

**Variate**	**Item**	**CITC**	**Cronbach's alpha if item deleted**	**Cronbach's alpha**
Athlete identity	—	—	—	0.820
Social identity	ai1	0.649	0.778	0.762
	ai2	0.572	0.790	
	ai3	0.616	0.782	
Exclusivity	ai4	0.507	0.806	0.758
	ai5	0.684	0.769	
Negative affectivity	ai6	0.461	0.822	0.684
	ai7	0.539	0.796	
Family life quality	—	—	—	0.932
Family interaction	flq1	0.633	0.928	0.852
	flq2	0.532	0.930	
	flq3	0.679	0.927	
	flq4	0.726	0.926	
	flq5	0.647	0.928	
	flq6	0.593	0.929	
Parenting	flq7	0.482	0.931	0.793
	flq8	0.528	0.930	
	flq9	0.514	0.930	
	flq10	0.615	0.928	
	flq11	0.602	0.929	
	flq12	0.641	0.928	
Emotional health	flq13	0.587	0.929	0.784
	flq14	0.685	0.927	
	flq15	0.696	0.927	
	flq16	0.675	0.927	
Physical wellbeing	flq17	0.647	0.928	0.821
	flq18	0.539	0.930	
	flq19	0.635	0.928	
	flq20	0.494	0.930	
	flq21	0.616	0.928	

### 2.4 Data analysis

**Figure 2:**
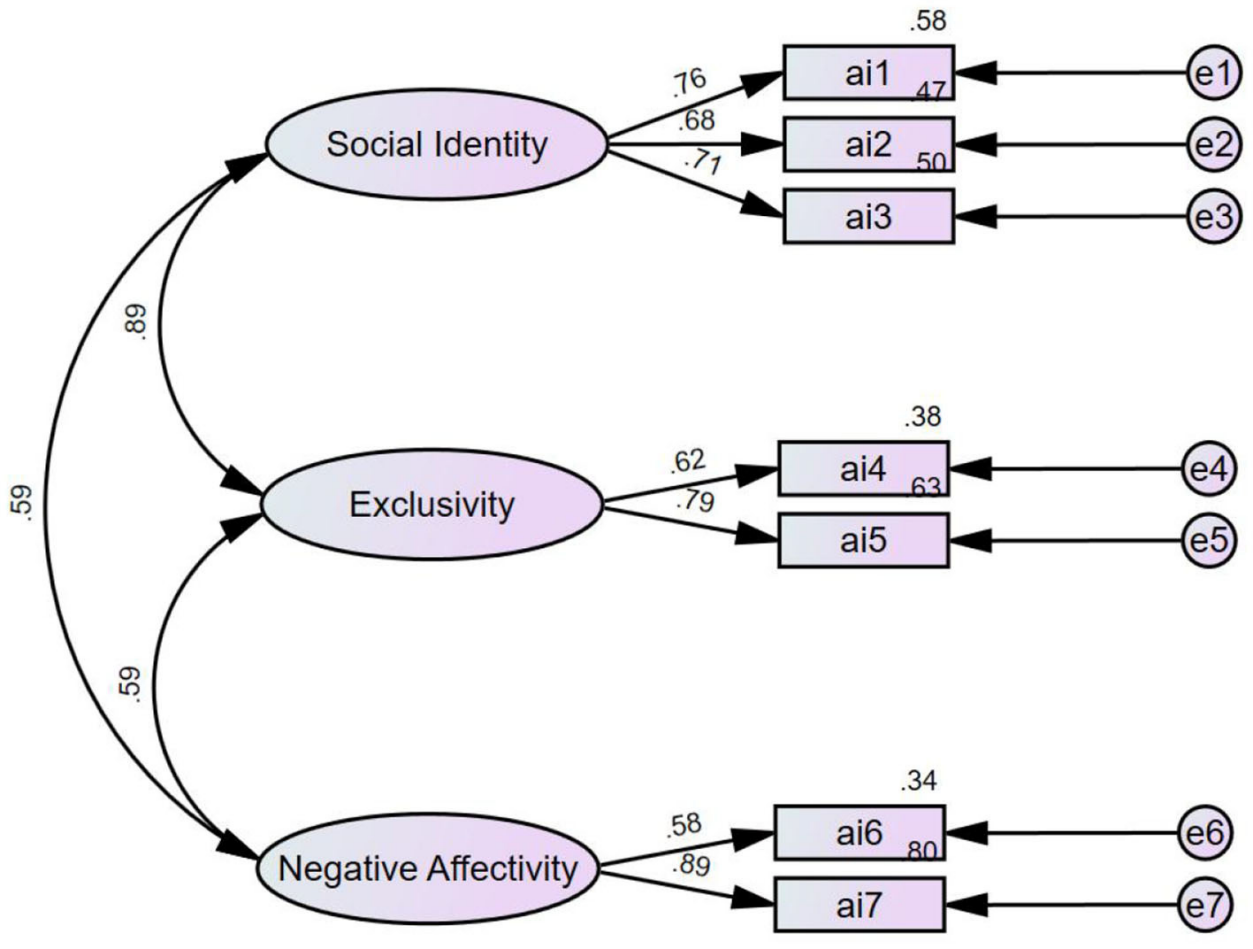
CFA model of athlete identity.

Confirmatory factor analysis: Figure 2 shows the validation CFA model for AMOS version 26 of the Athlete Identity Scale. To determine the validity of the validated CFA model, this study measured it by measuring the goodness of Fit index.

**Figure 3:**
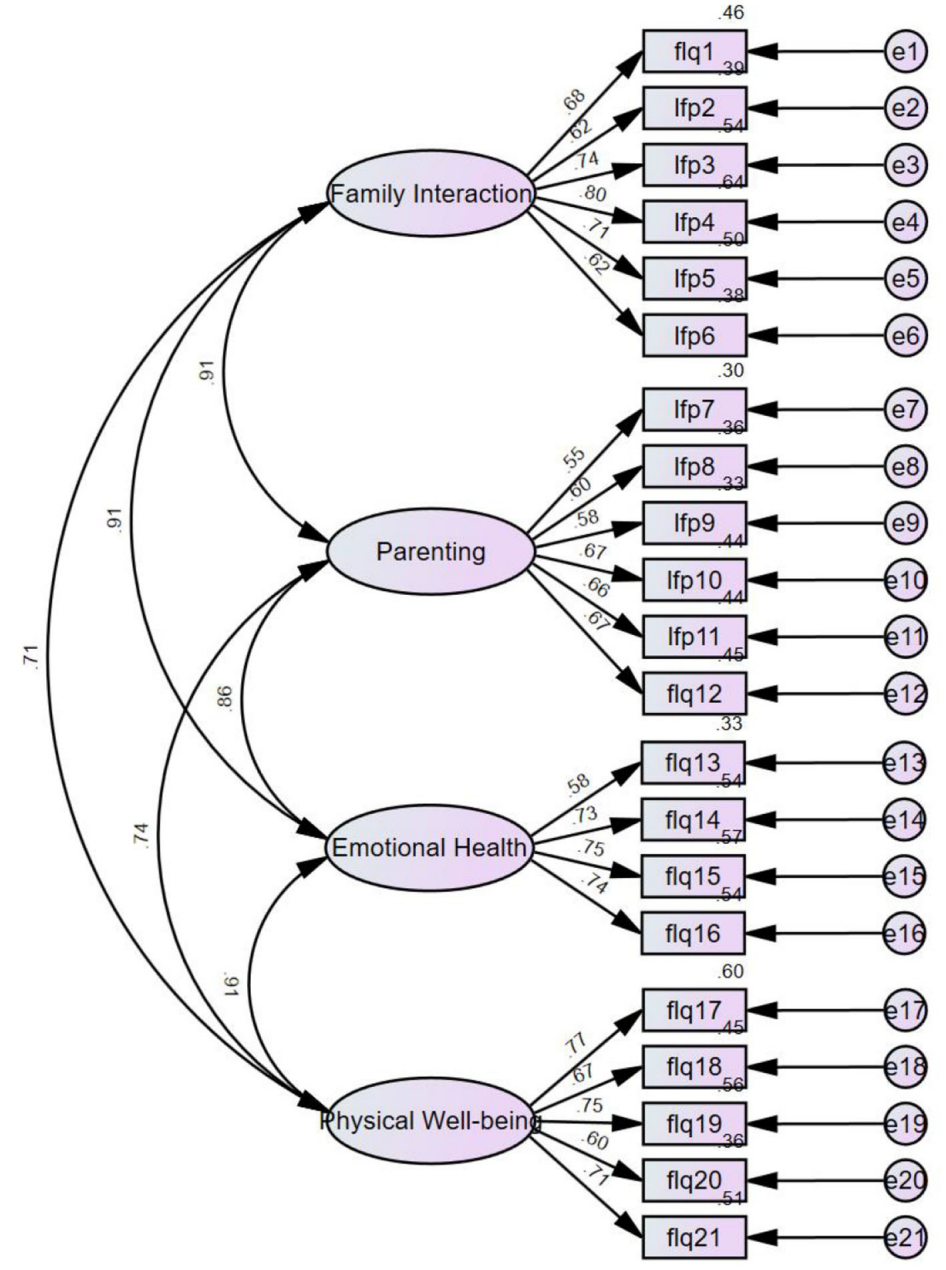
CFA model of family life quality.

Likewise, by employing the CFA to measure the validity of the Family Life Quality Scale, the degree of correlation between the schematic's four-dimensional frameworks is depicted in Figure 3.

Further structural validity checks were assessed using confirmatory factor analysis (CFA) for the well-translated and modified questionnaires. The following fitting indexes are commonly used to evaluate the fitting degree of a model: (a) Discrepancy Divided by Degree of Freedom(CMIN/DF). If the CMIN/DF value is ≤ 3, it indicates an acceptable fit; if the value is ≤ 5, it indicates a reasonable fit (Marsh and Hocevar, 1985). (b) Standardized Root Mean Square Residual (SRMR). It is the average difference between the observed correlation matrix (which represents the perfect prediction) and the matrix predicted by the model. Values <0.08 are generally considered indicative of a good fit. (c) Comparative Fit Index (CFI) and Tucker-Lewis Index (TLI): both these indices compare the fit of the specified model to the null model. Values close to 0.95 or above are generally considered indicative of a good fit. (d) GFI stands for Goodness of Fit Index and is used to calculate the minimum discrepancy function necessary to achieve a perfect fit under maximum likelihood conditions (Tanaka and Huba, 1985). GFI takes values of ≤1 where 1 represents a perfect fit.

The goodness-of-fit index of the data in Table 2 shows that the validity of the Athlete Identity Scale is relatively reasonable, and the ternary structure of the scale (social identity, exclusivity, negative affectivity) matches the structure of the sample data. It can also be seen that the validity of the family life quality scale is reasonable, and the four-element structure of the scale (family interaction, parenting, emotional health, physical wellbeing) matches the structure of the sample data.

**Table 2:** Goodness of fit indices for athlete identity and family life quality.

**Scale**	**CMIN/DF**	**SRMR**	**CFI**	**TLI**	**GFI**
Athlete identity	2.66	0.06	0.92	0.85	0.93
Family life quality	1.68	0.07	0.86	0.87	0.80

Finally, the composite reliability (CR) and convergence validity (AVE) tests were conducted for all scale items, and the results were shown in the Table 3. CR measures how well each latent variable is consistently explained by all the measurement items that make up that latent variable. The average variance extracted (AVE) value should be at least 0.50 or above, however, AVE value of more than 0.40 is acceptable if the composite reliability CR value is adequate (Maruf et al., 2021). Table 3 shows that all measures in each potential variable consistently describe the potential variable and show strong convergence validity.

**Table 3:** Reliability of all the factors.

**Variate**	**Item**	**Standardized estimate**	**CR**	**AVE**	***P***
Social identity	ai1	0.764	0.763	0.518	
	ai2	0.684			<0.001
	ai3	0.708			<0.001
Exclusivity	ai4	0.618	0.669	0.506	
	ai5	0.794			<0.001
Negative affectivity	ai6	0.582	0.715	0.567	
	ai7	0.892			<0.001
Family interaction	flq1	0.621	0.833	0.458	—
	flq2	0.735			<0.001
	flq3	0.798			<0.001
	flq4	0.710			<0.001
	flq5	0.616			<0.001
	flq6	0.551			<0.001
Parenting	flq7	0.603	0.795	0.394	—
	flq8	0.578			<0.001
	flq9	0.667			<0.001
	flq10	0.663			<0.001
	flq11	0.669			<0.001
	flq12	0.576			<0.001
Emotional health	flq13	0.754	0.837	0.562	—
	flq14	0.733			<0.001
	flq15	0.737			<0.001
	flq16	0.774			<0.001
Physical wellbeing	flq17	0.750	0.804	0.453	—
	flq18	0.672			<0.001
	flq19	0.598			<0.001
	flq20	0.711			<0.001
	flq21	0.621			<0.001

Kolmogorov-Smirnov Test (KS Test) was used to test the data distribution, and it was found that the sample data was not normally distributed. Therefore, the non-parametric Spearman order correlation is used for preliminary correlation analysis. Then, the structural equation model (SEM) was used for the mediation analysis (Hayes, 2018). All statistical analyses were performed with IBM SPSS Statistics for Windows, Version 27.0.

## 3 Results

### 3.1 Preliminary analyzes

According to the statistics of the survey data, there are 25 Level I athletes and 22 Level II athletes in the sample, and the number of people who have obtained the athlete grade certificate accounts for 42.3%. Male athletes account for 60% of the total.

Demographic information such as athlete's age and gender was entered in the preliminary analysis and did not demonstrate any relationship with the predictor or outcome variables. Thus, these two items were not highlighted in subsequent analyses. Spearman correlation coefficient showed that athlete identity was correlated with family life quality (r = 0.392, *P* < 0.001); athlete achievement was correlated with athlete identity (r = 0.463, *P* < 0.001) and family life quality (r = 0.248, *P* < 0.001); and family life quality. All other specific correlations are shown in Table 1.

It is not difficult to find that the athlete rating certificate has significant correlation with several dimensions of athlete identification and family life quality, except for the parenting dimension in the family quality of life scale. But, negative affectivity has no significant correlation with family interaction, parenting, emotional wellbeing and physical wellbeing. The items with the highest average scores are, in order: Negative affectivity (M = 4.18); Physical Wellbeing (M = 3.88); Family Interaction (M = 3.80). The lowest average scores were exclusivity (M = 3.44).

This article tested the influence of family life quality on athlete achievement, mediated by athletes identity. The results showed that family quality of life had a significant positive predictive effect on athlete achievement (β = 0.272, *P* < 0.05), and it had a significant positive predictive effect on athlete identification (β = 0.436, *P* < 0.001). When family quality of life and athlete identity enter the regression equation at the same time, the positive predictive effect of family quality of life on athletes' achievement is not significant (β = 0.097, *P* > 0.05), and the positive predictive effect of athlete status on athletes' performance is significant (β = 0.402, *P* < 0.001) (see Table 2).

Then, the intermediate effect test and confidence interval calculation were carried out by Bootstrap method: neither the indirect effect [0.078, 0.309] nor the total effect [0.074, 0.469] included 0 in the 95% confidence interval, and the direct effect [−0.106, 0.299] included 0 in the 95% confidence interval. That is, the total effect is significant, and the indirect effect mediated by athlete identity was also significant, but the direct effect of family life quality on athlete achievement is not significant, that is, complete mediator. Among them, the indirect effect accounted for 64.6%. Other control variables (i.e., athlete's age and gender) did not demonstrate any significant effect in the model (see Table 3).

### 3.2 Main findings

(a) Family quality of life is a positive predictor of athlete achievement (β = 0.272, *P* < 0.05). In addition, there was a significant correlation between family quality of life and athletes' achievement (r = 0.248, *P* < 0.001). However, the correlation between parenting and athlete achievement was not significant. The correlation coefficients of physical wellbeing, emotional wellbeing and family interaction dimensions were significant. Secondly, these athletes who still insist on participating in professional badminton training in high school generally have a better family material foundation and a harmonious family interaction atmosphere, but they are slightly inferior in terms of emotional support (see Table 4).
(b) Table 5 shows that family quality of life is a positive predictor of athlete identity (β = 0.436, *P* < 0.001). And there was a significant correlation between family quality of life and athlete identity (r = 0.392, *P* < 0.001). Athletes' negative affectivity are typical of athletes' identity, regardless of their skill level, they will be affected by sports injuries and terrible sports performance, resulting in negative emotions such as despair and pain. This kind of negative affectivity cannot be effectively alleviated by family interaction, parenting, material conditions, and emotional support, and the appearance of negative emotions cannot be avoided regardless of the quality of family life.
(c) Athlete identity plays a mediating role between family quality of life and athlete achievement. Table 6 shows that direct effects (0.097) and intermediate effects (0.175) account for 35.4% and 64.6% of the total effects (0.271), respectively. Although the data show that it is fully mediated, it is of little practical significance to distinguish between full mediation and partial mediation from a practical point of view. In addition to athlete identity athlete achievement must consider the comprehensive effect of coaches, peers, schools, society and other aspects.
(d) Men still dominate badminton sports. Although data analysis showed that there was no significant correlation between gender and athlete achievement, male athletes had more opportunities to earn a rating certificate than female athletes, which was related to the higher number of male athletes. Almost all badminton training teams have more men than women. Male athletes accounted for 56.8% (*n* = 63), the proportion of male athletes with athlete level certificates was 61.7% (*n* = 29).

**Table 4:** Spearman correlations table.

**Variable**	**Mean**	**SD**	**AA**	**1**	**2**	**3**	**4**	**5**	**6**
1.Social identity	3.68	0.93	0.461^**^						
2.Exclusivity	3.44	1.16	0.322^**^	0.645^**^					
3.Negative affectivity	4.18	0.93	0.269^**^	0.425^**^	0.340^**^				
4.Family interaction	3.80	0.86	0.191^*^	0.340^**^	0.412^**^	0.131			
5.Parenting	3.72	0.84	0.091	0.301^**^	0.260^**^	0.009	0.720^**^		
6.Emotional wellbeing	3.62	0.99	0.300^**^	0.441^**^	0.418^**^	0.158	0.771^**^	0.697^**^	
7.Physical wellbeing	3.88	0.87	0.316^**^	0.419^**^	0.359^**^	0.089	0.593^**^	0.563^**^	0.703^**^

AA, Athlete Achievement.

* *p* < 0.05;

** *p* < 0.001.

**Table 5:** Mediator effect test.

**Outcome predictor variable**	**Predictive outcome variable**	**Fit Index**		**Coefficient significance**	
		**R** ^2^	**F**	β	**t**
FQOL	AA	0.253	7.437	0.272	2.727^*^
FQOL	AI	0.170	22.274	0.436	4.720^**^
FQOL	AA	0.194	12.973	0.097	0.948
AI				0.402	4.170^**^

AI, Athlete Identity; AA, Athletes' Achievement; FQOL, Family Quality of life.

* *p* < 0.05;

** *p* < 0.001.

**Table 6:** Analysis of mediating effects.

**Variable**	**Effect size**	**Boot SE**	**95%CI**	**Effect ratio (%)**
Direct effect	0.097	0.102	(−0.106, 0.299)	35.4
Indirect effect	0.175	0.060	(0.078, 0.309)	64.6
total effect	0.271	0.100	(0.074, 0.469)	100

## 4 Discussion

The results of the study support the three hypothesis: H_1_: Family life quality positively influences the achievement of young badminton athletes; H_2_: Athlete identity mediates the relationship between family life quality and badminton athletic achievement of young athletes; H_3_: The quality of family life positively affects the identity of young badminton athletes.

First of all, from the impact of family life quality on young badminton players, the most important factors are emotional wellbeing and physical wellbeing, and the physical wellbeing is slightly higher than the emotional wellbeing. In other words, athletes need material support more than emotional support and understanding. This may be related to the high consumption of badminton itself. These training conditions are inseparable from the support of material foundation and social resources. In the family-oriented Chinese society, parents and other family members cannot sit idly by and ignore the athletes' pleas for help. Therefore, the quality of life at home directly or indirectly affects the quality and effectiveness of the training received by the athlete, which is manifested through the athlete's achievement. Family interaction can also give athletes the strength to identify with their basic training requirements.

Secondly, the mediating effect of the athlete's identity on the achievement of adolescent badminton cannot be ignored. And data show that athlete identity is more correlated with athlete achievement than family quality of life (r = 0.463 > 0.248, *P* < 0.001). That is to say, when the major entities focus on the investment in the achievements of athletes, it must be in moderation. Besides, intangible psychological care is more important than tangible material care. Most families in China are lacking in this area, and they are shy to express love and care, replacing them with harshness and reprimands. Especially after China has fully entered a moderately prosperous society, the material conditions of athletes' families have generally been guaranteed, but the spiritual care has not been improved at the same time (M = 3.88 > 3.62, *P* < 0.001).

Finally, from the perspective of the influence of family life quality on athlete identity, this influence is also positive and significant. That is, the higher the quality of family life, the stronger the identity of badminton players. Finally, from the perspective of the influence of family life quality on athlete identity, this influence is also positive and significant. The higher the quality of family life, the stronger the identity of badminton players. But let's face it, the quality of family life can't do much about it when it comes to negative emotions, that is the negative emotions in competitive competition or training need to be digested and overcome by the athletes themselves.

The researchers explored and analyzed the main factors influencing athletes' achievement from various angles. It tells parents that too much attention and worry is unnecessary, and it is even more unreasonable to give up their normal work and life for their children's achievements. These overloads of care and dedication are a constraint and pressure for athletes, which can affect their athletic achievements. In addition, the current family care for the athlete's soul is not enough, and harmonious family interaction and close parenting have not yet become a common social phenomenon.

### 4.1 Implications

Previous research has emphasized addition, thinking about what parents can do for athlete achievement. This article emphasizes subtraction, reducing some unnecessary over-effort from the athlete's point of view. Although some studies have pointed out that athletes' perceptions determine the positive or negative effects of parental support (Flammer, 1995; Rouquette et al., 2021). This article emphasizes that parents should focus on intangible aspects of communication and support such as psychological level and family interaction, so as to give athletes more freedom in sports. After all, the results of the study cannot be denied, for athletes, there are still some aspects of family quality of life that cannot be interfered with and avoided.

Furthermore, the purpose of today's parents and badminton players participating in sports is too utilitarian. They insist that the purpose of training and competition is to further education and employment, not to simply love the sport they do. Therefore, when their efforts do not achieve the desired rewards and achievements, they will feel anxious, uneasy and stressed. These negative emotions in turn can affect their competitive status, thus creating a vicious circle. At the same time, it is also contrary to the original intention of sports. Participation in sports should play a role in strengthening the body, venting emotions and tempering the will, so as to achieve all-round human development. However, the excessive pursuit of medals and honors completely limits the functioning of sports, and on the contrary, it leads to sports injuries, emotional stress and willpower breakdown. This article advocates sports participation that keeps oneself and loves sports, and builds a strong body while working hard to complete one's studies, rather than seeing it as a shortcut to further education.

### 4.2 Limitations

The subjects of this study are mainly from Hunan Province in central China, whose strong badminton culture and professional training experience attract more young people to engage in badminton. And the economic level of the region is at the forefront of the country. These factors will affect the achievements of young badminton players, so the conclusions of this study cannot be a general summary, but can provide reference for other areas of research. Perhaps young athletes in more economically developed areas have more pure sports goals, and they are more likely to achieve their own satisfaction in sports achievements. In addition, badminton is only one type of sports, and whether athletes in other sports, such as martial arts, tennis, football, basketball, etc., have the same athlete identity, and whether their family quality of life also affects their sports achievements, needs to be further studied. Similarly, the care input of the main body in the athlete environment, such as coaches and school teachers, should take into account the principle of proportionality, and the necessity of such care should be considered from the perspective of the athlete.

## 5 Conclusion

This study focuses on the impact of family on athlete achievement through a quantitative research lens. The results of this study suggest that the quality of family life has a positive impact on athletes' achievement, and athlete identity plays a mediating effect. A better economic and material basis is the general family quality of life characteristic of these successful athletes. Emphasizing material things over psychology is a common characteristic of these families. However, the negative affectivity of athlete identity is a factor that affects the athlete's achievement that every athlete needs to digest and cannot be directly intervened and solved by the family. This research findings have important reference value for parents' concept of education and coach management. To be specific, coaches should fully conduct a comprehensive investigation of their family conditions when selecting the main badminton players, including family economic base, parental rearing style, parental emotional support and family sports atmosphere. Families who perform well in these aspects are more likely to cooperate with the coach's training requirements and plans, and thus have better sports performance and achievements. For parents, cultivating excellent athletes should give more emotional support and practical companionship in ensuring the basic material conditions. And these support should be moderate, too much care will cause mental pressure on the athletes, affect their play. The most important point is to allow athletes to have negative downturns and accept their failures, which is especially important for Chinese parents. For athletes, accepting the attention of their families and focusing on their own training is a better choice to achieve sports achievements. Learning to adjust your mind and relieve stress is the key to winning on the court, but also an important lesson in the long road of life. This article advocates pure, simple purpose-oriented participation in sports, and advocates the realization of dual care for the body and mind in sports. This article suggests that parents give athletes more freedom to grow and not pay too much attention to their children's own affairs.

## Data Availability

The raw data supporting the conclusions of this article will be made available by the authors, without undue reservation.
